# Establishment and validation of a prognostic nomogram based on a novel five‐DNA methylation signature for survival in endometrial cancer patients

**DOI:** 10.1002/cam4.3576

**Published:** 2020-12-22

**Authors:** Xingchen Li, Fufen Yin, Yuan Fan, Yuan Cheng, Yangyang Dong, Jingyi Zhou, Zhiqi Wang, Xiaoping Li, Jianliu Wang

**Affiliations:** ^1^ Department of Obstetrics and Gynecology Peking University People's Hospital Beijing China; ^2^ Beijing Key Laboratory of Female Pelvic Floor Disorders Diseases Beijing China

**Keywords:** DNA methylation, endometrial cancer, nomogram, risk model

## Abstract

**Background:**

This study aimed to explore the prognostic role of DNA methylation pattern in endometrial cancer (EC) patients.

**Methods:**

Differentially methylated genes (DMGs) of EC patients with distinct survival from The Cancer Genome Atlas (TCGA) database were analyzed to identify methylated genes as biomarkers for EC prognosis. The Least Absolute Shrinkage and Selection Operator (LASSO) analysis was used to construct a risk score model. A nomogram was built based on analysis combining the risk score model with clinicopathological signatures together, and then verified in the validation cohort and patients in our own center.

**Results:**

In total, 157 DMGs were identified between different prognostic groups. Based on the LASSO analysis, five genes (GBP4, OR8K3, GABRA2, RIPPLY2, and TRBV5‐7) were screened for the establishment of risk score model. The model outperformed in prognostic accuracy at varying follow‐up times (AUC for 3 years: 0.824, 5 years: 0.926, and 7 years: 0.853). Multivariate analysis identified four independent risk factors including menopausal status (HR = 3.006, 95%CI: 1.062–8.511, *p* = 0.038), recurrence (HR = 2.116, 95%CI: 1.061–4.379, *p* = 0.046), lymph node metastasis (LNM, HR = 3.465, 95%CI: 1.225–9.807, *p* = 0.019), and five‐DNA methylation risk model (HR = 3.654, 95%CI: 1.458–9.161, *p* = 0.006) in training cohort. The performance of the nomogram was good in the training (AUC = 0.828), validation (AUC = 0.866) and the whole cohorts (AUC = 0.843). Furthermore, we verified the nomogram with 24 patients in our center and the Kaplan–Meier survival curve also proved to be significantly different (*p* < 0.01). The subgroup analysis in different stratifications indicated that the accuracy was high in different subgroups for age, histological type, tumor grade, and clinical stage (all *p* < 0.01).

**Conclusions:**

Briefly, our work established and verified a five‐DNA methylation risk model, and a nomogram merging the model with clinicopathological characteristics to facilitate individual prediction of EC patients for clinicians.

## Graphical abstract

In this study, we revealed a five‐DNA risk model based on methylation profiling based on TCGA database. The prognostic accuracy for 5‐year survival reached to 0.926 by AUC. Furthermore, we established and validated an EC prognostic nomogram consisting of five‐DNA methylation risk model and clinicopathological characteristics, and the nomogram outperformed in training (AUC = 0.828) and validation (AUC = 0.866) cohorts. This nomogram could also be expected to serve as a predictive tool for patients in different subgroups.

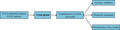

**Abbreviations**
AUC: area under the ROC curve
C‐index: concordance index
DMGs: differentially methylated genes
EC: endometrial cancer
FDR: false‐discovery rate
FIGO: international federation of gynecology and obstetrics
GSEA: gene set enrichment analysis
HR: hazard ratio
LASSO: least absolute shrinkage and selection operator
OS: overall survival
TCGA: The cancer genome atlas
tdROC: time‐dependent receiver operating characteristic
WGCNA: weighted correlation network analysis

## 1 BACKGROUNDS

Endometrial cancer (EC) is a common tumor for women both in China and worldwide.^1^ Irregular vaginal bleeding after menopause is the typical symptom of EC, and it is often found in the early stage of the disease course. Therefore, patients diagnosed at an early stage have a favorable prognosis and the 5‐year survival rate of EC patients is greater than 85%. However, approximately 13–25% of EC patients with late stage will lead to metastasis or recurrence.^2^ Complex biological processes and unintelligible molecular mechanism often lead to unfavorable prognosis in EC patients. Hence, there is an urgent need to explore the molecular mechanism of EC and identify novel targets for treatment and intervention for EC patients.

Epigenetic dysregulation is an essential mechanism in tumorigenesis that affects numerous gene expressions.^3^ Among all the epigenetic formation, DNA methylation is an important epigenetic way. Proper DNA methylation is necessary during cellular growth and development. However, due to its role in the regulation of genomic transcription, aberrant methylated genes can also lead to a series of diseases including cancer.^4^ Many studies have shown that abnormal DNA methylation is related to irregular gene silencing, genome stability, and cell fate determination.^5^ Abnormal DNA methylation, including hyper‐ and hypo‐methylation, on CpG islands of promoters is one such essential mechanism. There are evidences that aberrant DNA methylation frequently occurs in early development of tumors,^6^ and these alterations are relatively stable and potentially reversible therapeutically.^7^ Therefore, the dysregulated DNA methylated status serves as a perfect biomarker for clinical diagnosis and decision making for clinicians.

In pursuit of predictive factors for patients with EC, an increasing number of studies have identified some predictive biomarkers. In one study, He et al. reported that the methylation related to DNMT3A/3B overexpression might play a vital role in ER/PR downregulation, thus leading to poor prognosis for EC patients.^8^ Another study indicated that the altered DNA methylation of four genes, including ZNF154, PCDHs, TNXB, and DPP6 could predict the overall survival (OS) of EC and guide the personalized therapy.^9^ Therefore, in order to find novel biomarkers for the survival of EC patients, it is reasonable to analyze the DNA methylation pattern in tumor cells.

Despite the extensive studies on the relationship between abnormal DNA methylation and the prognosis of EC patients, individualized prognostic nomogram considering both methylated features and clinical characteristics had rarely been reported. In this study, we identified a set of OS‐related DNA methylation and established a risk model by least absolute shrinkage and selection operator (LASSO) analysis using the data from TCGA database. Furthermore, we constructed a nomogram through integrating methylation expression profiling and clinicopathological indexes to predict the OS. The independence and repeatability of this nomogram as a prognostic signature were verified in the validation cohort, the whole cohort, and our own patients.

## 2 METHODS

### 2.1 Data acquisition and procession

The DNA methylation data and corresponding clinicopathological features (FIGO stage, tumor grade, histological type, recurrence, peritoneal cytology, and lymph node metastasis) were retrieved form TCGA (https://portal.gdc.cancer.gov/) database. We deleted the patients with incomplete clinicopathological data. The DNA methylation expression was scored using β‐values ranging from 0 (unmethylated) to 1 (completely methylated, Infinium HumanMethylation450 BeadChip), and beta‐value was calculated as the ratio of M and M + U, where M represents the signal from methylated beads targeting CpG site, while U represents the signal from unmethylated beads. We divided the patients into two groups according to their overall survival (OS), OS <1 year and >3 years. *DESeq* and *limma* package of R software was used to identify differentially methylated genes (DMGs) analysis between the two groups.^10^, ^11^ We used the false‐discovery rate (FDR) to adjust the *P*‐values obtained by the Mann–Whitney U test. An absolute log_2_‐fold change (|log_2_FC|) > 1 and an adjusted *p* < 0.05 were used for screening DMGs. In our own testing cohort, 24 EC samples with RNAseq expression and clinical data were obtained from surgically treated patients were acquired in our center. We transferred the RNAseq data into methylated expression according to the formula we constructed by analyzing TCGA database. These methylated data were used for further validation with different risk groups. This study was approved by the Institutional Ethics Committee (Human Research) of Peking University People's Hospital.

### 2.2 Construction of the risk predictive model

We brought the genes appeared in both groups into a LASSO regression analysis to narrow the range of DMGs. This method was popular in machine learning and implemented by the R package “*glmnet*” and could avoid overfitting of the prognostic model.^12^ The selected DMGs were screened more than 1000 times, and if specific DMGs were detected more than 800 times, they were regarded as candidate biomarkers. Furthermore, they were analyzed by multivariate Cox regression analysis designed to identify the hub genes by controlling confounding factors or covariates. The selected genes resulted from LASSO analysis was used to construct a risk signature. The risk score based on the signature of each patient was calculated using the following formula^13^:

$$\text{Risk score}=\sum n_{i}=\sum \left(\text{Corfi}*x_{i}\right)$$

Coefi was the coefficient and x_i_ was the z‐score transformed relative expression value of each selected gene. We calculated the score of each patient and categorized the whole population into high‐ and low‐risk groups according to the cut‐off value of risk score, and then built a risk predictive model. Time‐dependent receiver operating characteristic (tdROC) curve analysis and survival curve were plotted to evaluate the predictive accuracy of the risk model for OS based on the risk score model.

### 2.3 Bioinformatics analysis

To compare the expression of DMGs between shorter and longer survival time, the R package “*pheatmap*” were used. In order to explore the role of the selected methylated genes from LASSO analysis, we explored the correlation among these selected genes, and the relationship between gene expression and methylation level in EC patients. A gene co‐expression network was built by the Weighted Gene Correlation Network Analysis (WGCNA) using DEGs (Differentially Expressed Genes) between normal and cancer samples.^14^ We first filtered out the best soft threshold by “*WGCNA*” R package to maintain sufficient connectivity and keep the gene network close to the scale‐free topology. Furthermore, we evaluated the correlation between the risk and the modules to identify the key module. GO and KEGG of the key module resulted from WGCNA analysis were performed by Metascape (metascape.org).^15^ These significant functional terms were then hierarchically clustered into a tree based on κ‐statistical similarities among their gene memberships. We used 0.3κas the threshold to organize the tree in Metascape. Gene Set Enrichment Analysis (GSEA) was conducted to study the functions associated with different risk groups of EC. Regarding the GSEA results, |NES| > 1 and nom *p*‐value <0.05 were considered significant in our study.

### 2.4 Development and validation of the nomogram

To further evaluate whether the risk factor classifier was independent risk factors for OS, we divided all of the patients into two cohorts, the training cohort and the validation cohort. We carried out univariate and multivariate Cox regression analyses with risk score model in the two cohorts and combined the latter with other clinicopathological characteristics to construct a nomogram with these independent prognostic factors using “*rms*” package in the training cohort.^16^ Calibration curves were drawn and the concordance index (C‐index) was computed to assess the accuracy of the nomogram in both cohorts. The prognostic risk value of each patient was calculated using the nomogram and the whole group was then evenly classified into three subgroups including high‐, moderate, and low‐score subgroups. The prognostic evaluation of nomogram was then performed with Kaplan–Meier survival analysis. This nomogram was also validated in the 24 patients in our center. To further validate the predictive efficiency of the risk score model of the risk model, we compared the area under the tdROC curve (AUC) of the combined independent factors with or without the risk model. A subgroup analysis was performed by classifying the patients into different clinicopathological stratifications (age, histological type, tumor grade, and clinical stage) and then analyzed the survival by low‐, moderate‐, and high‐score subgroups. The survival estimation and ROC curve of patients were analyzed with the Kaplan–Meier method by “*survival*” and “*survivalROC*” package in R. In addition, *p*‐value <0.05 was considered statistically significant.^17^

### 2.5 Statistics

Two‐tailed Student's *t*‐test or one way ANOVA test was used for statistical comparison between different groups. The correlation between mRNA expression level and DNA methylation level was analyzed for every gene by Spearman correlation coefficient. Univariate and multivariate Cox regression analyses were applied to identified the hazard ratio (HR), prognostic factors, and different clinicopathological characteristics. All statistical analyses were performed by R software (R version 3.6.1). *p*‐value <0.05 was considered statistically significant.

## 3 RESULTS

### 3.1 Characteristics of EC patient in datasets

**FIGURE 1:**
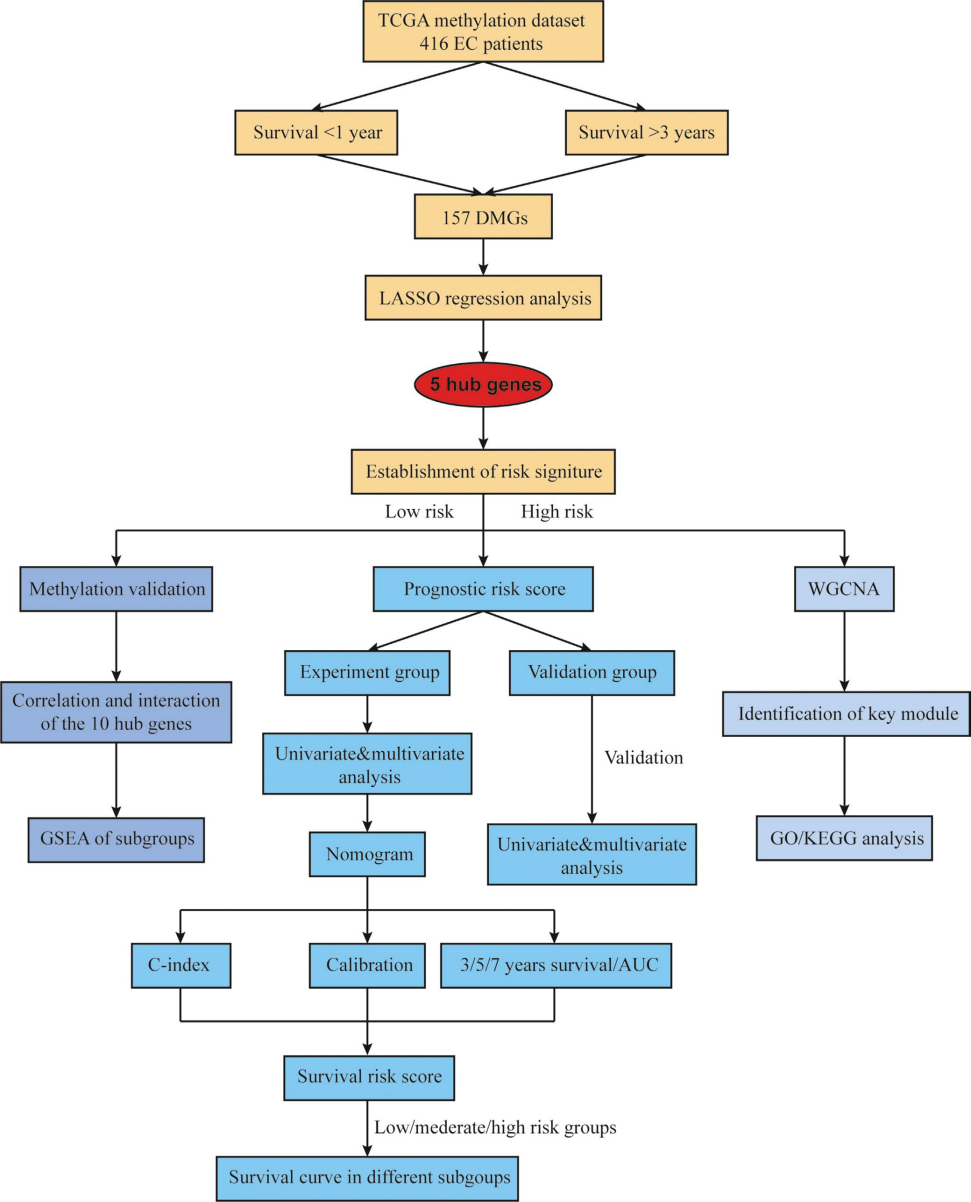
The flow chart of the study design and analysis

In total, we extracted 416 patients with EC from the TCGA database who had been diagnosed clinically and pathologically, and these data were analyzed as the flowchart (Figure 1). According to the overall survival (OS), there were 33 patients whose OS was less than 1 years, 138 patients whose OS was more than 3 years, and the rest (245 patients) were between 1 to 3 years. The patients were then randomly categorized into two cohorts, and each cohort consisted of 208 patients. The median age of the training cohort and the validation cohort was 64.7 ± 10.2 and 63.7 ± 11.8 years old. The median OS was 999.4 ± 774.3 and 1000.5 ± 929.7 days. Based on the pathological characteristics of FIGO stage, tumor grade, histological type, and LNM, the majority of the patients in the two cohorts were both diagnosed with stage I (61.5% and 61.0%), G3 (61.1% and 66.8%), EEA (71.1% and 73.6%), and negative LNM (73.1% and 72.1%). There was no significant difference in all of the clinicopathological features between the two cohorts (all *p* > 0.05). The main basic and pathological characteristics of the two groups were illustrated in Table 1.

**TABLE 1:** Characteristics of patients in training and validation cohorts

Variables	Whole cohort	Training cohort	Validation cohort	*p*‐value
Total number	416	208	208	
Age (year)	64.20 ± 11.0	64.7 ± 10.2	63.7 ± 11.8	0.267
OS (day)	999.7 ± 854.5	999.4 ± 774.3	1000.5 ± 929.7	0.516
OS (year)	N (%)			0.627
<1 year	33 (7.9)	16 (7.7)	17 (8.2)	
1–3 years	245 (58.9)	124 (59.6)	121 (58.2)	
>3 years	138 (33.2)	68 (32.7)	70 (33.6)	
Living status				0.699
Alive	343 (82.45%)	173 (83.2%)	170 (81.7%)	
Dead	73 (17.55%)	35 (16.8%)	38 (18.3%)	
Diabetes				1.000
No	286 (68.75%)	143 (68.8%)	143 (68.8%)	
Yes	130 (31.25%)	65 (31.2%)	65 (31.2%)	
Hypertension				0.480
No	159 (38.22%)	76 (36.5%)	83 (39.9%)	
Yes	257 (61.78%)	132 (63.5%)	125 (60.1%)	
Menopausal status				0.421
Premenopause	66 (15.87%)	30 (14.4%)	36 (17.3%)	
Postmenopause	350 (84.13%)	178 (85.6%)	172 (82.7%)	
FIGO stage				0.920
Stage I	255 (61.30%)	128 (61.5%)	127 (61.0%)	
Stage II	42 (10.10%)	20 (9.6)	22(10.6)	
Stage III	95 (22.83%)	49 (23.6)	46 (22.1)	
Stage IV	24 (5.77%)	11 (5.3)	13 (6.3)	
Tumor grade				0.472
G1	61 (14.66%)	33 (15.9%)	28 (13.5%)	
G2	89 (21.39%)	48 (23.0%)	41 (19.7%)	
G3	266 (63.94%)	127 (61.1%)	139 (66.8%)	
Histological type				0.584
EEA	301 (72.36%)	148 (71.1%)	153 (73.6%)	
Other types	115 (27.64%)	60 (28.9%)	55 (26.4%)	
Recurrence				0.903
No	331 (79.57%)	165 (79.3%)	166 (79.8%)	
Yes	85 (20.43%)	43 (20.7%)	42 (20.2%)	
Peritoneal cytology				0.604
Negative	344 (82.69%)	174 (83.7%)	170 (81.7%)	
Positive	72 (17.31%)	34 (16.3%)	38 (18.3%)	
LNM				0.826
Negative	302 (72.60%)	152 (73.1%)	150 (72.1%)	
Positive	114 (27.40%)	56 (26.9%)	58 (27.9%)	

Abbreviations: EEA, endometrioid endometrial adenocarcinoma, FIGO, international federation of gynecology and obstetrics, G1/2/3, grade 1/2/3, LNM, lymph node metastasis; OS, overall survival.

### 3.2 Identification and functional analysis of DMGs in EC

**FIGURE 2:**
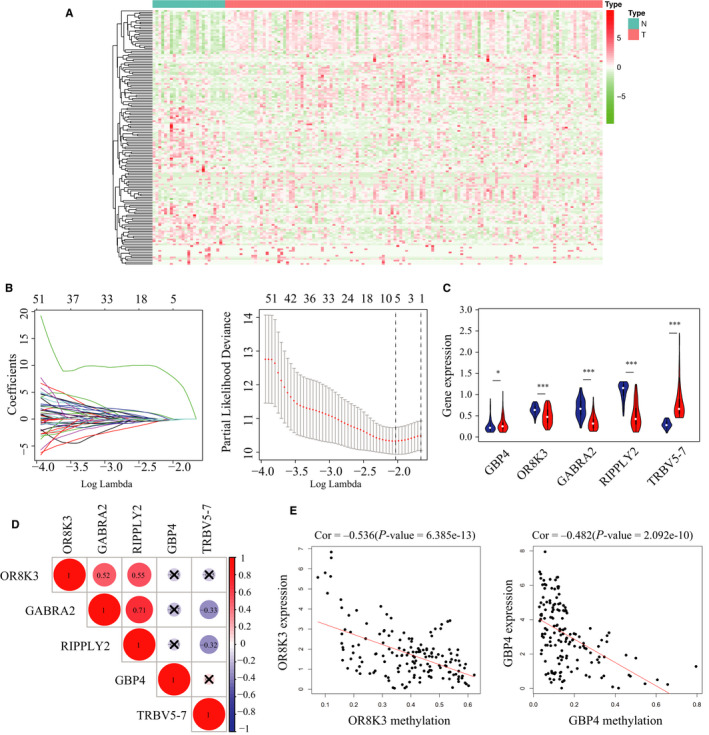
Identification of prognostic genes in EC patients. A, Heatmap of the expression of the 157 DNA methylation‐regulated genes. Wilcox test was used to determine the differential gene expression between the two groups. B, The process of selecting target genes in TCGA dataset with LASSO regression model. C, Expression of DNA methylation in normal and tumor tissues. The red fusiformis represents tumor tissue and the blue fusiformis represents normal tissue. D, The methylation relationship among five methylated genes. The bigger the circle size, the more correlative two genes are. E, Spearman correlation between gene expression and methylation of the OR8K3 and GBP4. Error bars represent the mean ± SD. *p < 0.05; **p < 0.01; ***p < 0.001

From the DNA methylation expression data in patients with EC, the DMGs between the patients whose OS less than 1 year and those whose OS more than 3 years, were screened and selected. A total of 157 DMGs associated significantly with the prognosis of EC patients were identified (Figure 2A). The results of the 157 DEGs were used in the LASSO regression to identify robust markers. To prevent overfitting, cross‐validation was carried out in five rounds. A group of five genes (GBP4, OR8K3, GABRA2, RIPPLY2, and TRBV5‐7) and the corresponding coefficients were computed (Figure 2B, Table 2). Compared with normal tissue samples, OR8K3, GABRA2, and RIPPLY2 were downregulated, while GBP4 and TRBV5‐7 were upregulated in EC tissue samples (Figure 2C). Our own sequencing data also proved this results. OR8K3, GABRA2, and RIPPLY2 were downregulated and GBP4, TRPV5‐7 were upregulated in lymph node metastasis, myometrial invasion and recurrence groups (Figure S2A–C). Then the interrelationships between the five DMGs were analyzed using the “*corrplot*” package in R software. The expressions of the five methylated DNA were positively associated with each other between GABRA2 and OR8K3, GABRA2 and RIPPLY2, OR8K3 and RIPPLY2 (Figure 2D). We also investigated the association between the expression of mRNA and methylation of the five genes. As shown in Figure 2E (and Figure S1), there is a significant inverse correlation between methylation and mRNA levels (|R| > 0.3, *p* < 0.05). These results indicated that these five genes might play an essential role in the prognosis of EC patients.

**TABLE 2:** Methylated genes and correlated coefficient value

Methylated gene	Coefficient
GBP4	0.139674
OR8K3	−0.01361
GABRA2	−0.21646
RIPPLY2	−0.21224
TRBV5‐7	8.09756
Risk score	Low: <1.07
	High: ≥1.07

### 3.3 Establishment of a risk score model based on the five methylated genes

**FIGURE 3:**
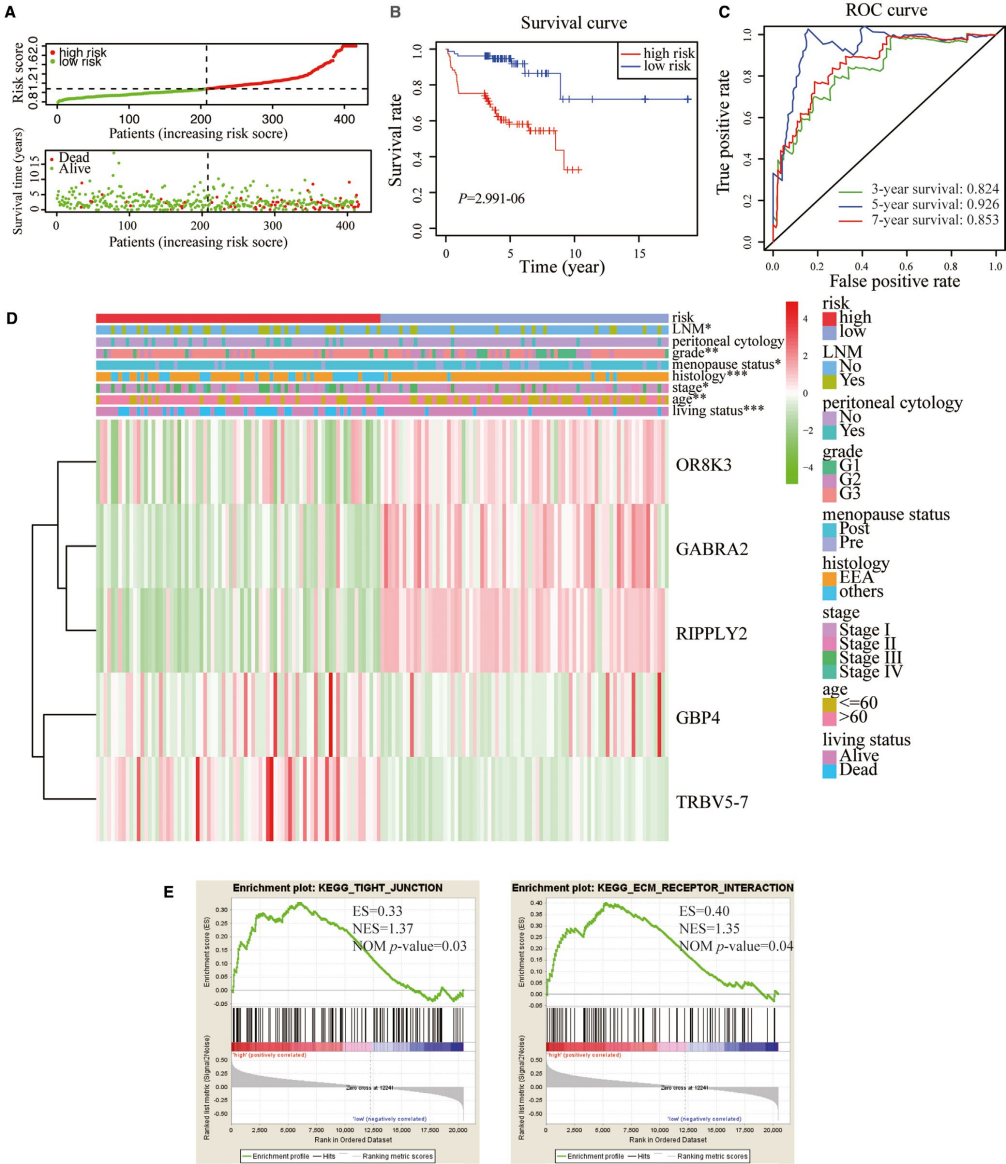
Construction and predictive accuracy in high‐ and low‐risk models and differential clinicopathological factors with TCGA dataset. A, Risk score distribution in the low‐ and high‐risk groups. B, Kaplan–Meier survival analysis of the low‐ and high‐risk groups. C, Time‐dependent ROC curves for 3‐, 5‐, and 7‐year survival prediction. D, Heatmap and clinicopathological features of the two groups. Chi‐square test was used for correlation between clinical and cluster, * *p* < 0.05, ** *p* < 0.01, and *** *p* < 0.001. E, GSEA results based on the risk model. GSEA results show that genes with higher expression in high‐risk group were enriched for hallmarks of malignant tumors in tight junction and ECM receptor interaction

Based on the coefficient value and expression of each methylated DNA, a risk score model was constructed. The predictive risk score was established by adding the product of the expression level and relative coefficient of each gene in the LASSO regression as follows: risk score = (0.139674*GBP4) + (−0.01361*OR8K3) + (−0.21646*GABRA2) + (−0.21224*RIPPLY2) + (8.09756*TRBV5‐7). In order to evaluate the prognostic role of the risk score model, each patient was calculated with a risk score, and we assigned the patients into high‐ and low‐risk groups according to the median risk score (Figure 3A). The Kaplan–Meier survival curve indicated that the survival of the low‐risk group patients was significantly better than that of the high‐risk group (*p* = 2.991‐06, Figure 3B). Patients in our center were also divided into two groups with this risk score formula, and Kaplan–Meier survival curve revealed significantly different between the two groups (Figure S3A). Methylated gene expression in two groups was shown with heatmap (Figure S3B). The AUC of the survival assessment model of the five methylated DNA was 0.824 of 3‐year, 0.926 of 5‐year, and 0.853 of 7‐year OS in TCGA dataset (Figure 3C). The expression levels of the five methylated DNA and the distribution of clinicopathological features in high‐ and low‐risk groups were presented in the heatmap (Figure 3D). The results revealed that there were significant difference between the high‐ and low‐risk groups in term of living status (*p* < 0.001), age (*p* < 0.01), menopausal status (*p* < 0.05), histological type (*p* < 0.001), clinical stage (*p* < 0.05), tumor grade (*p* < 0.01), and LNM (*p* < 0.05). To investigate the tumorigenesis mechanism of the gene function in high‐risk group, GSEA was performed to analyze the mRNA expression of 416 EC patients. As displayed in Figure 3E, we figured out that the five methylated DNA might play a pivotal role in tight junction (ES = 0.33, NOM *p* = 0.03) and ECM receptor interaction (ES = 0.40, NOM *p* = 0.04). The outcomes suggested that the risk score model might accurately predict the prognosis of EC patients.

### 3.4 Identification and bioinformatics analysis of the key module associated with risk model

**FIGURE 4:**
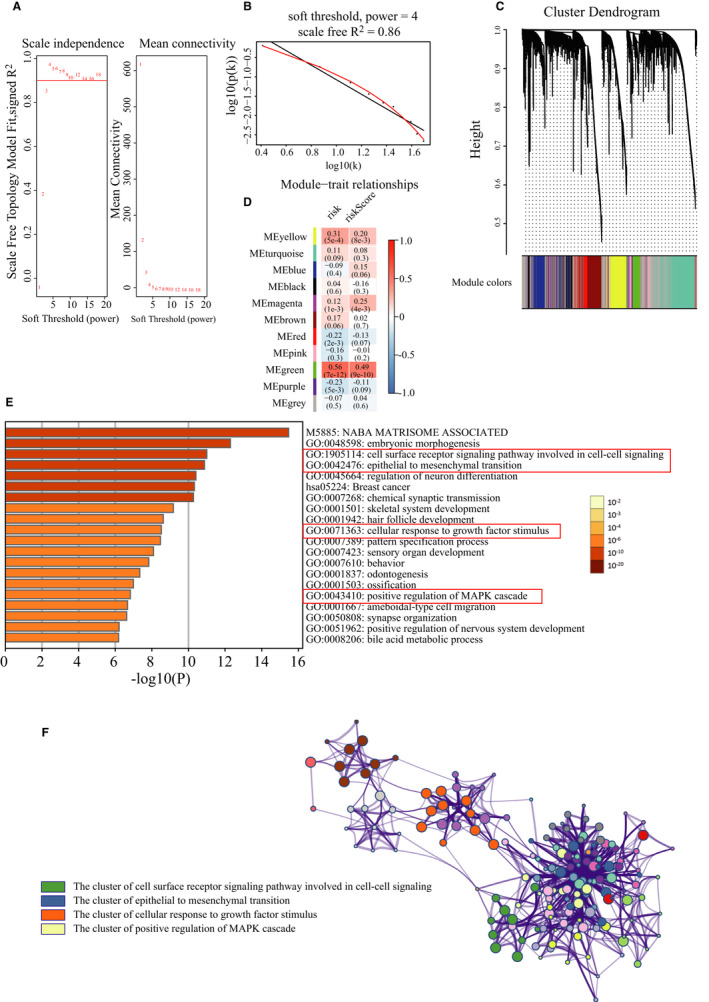
Results of weighted correlation network analysis (WGCNA) on the RNA‐seq database and selection of hub genes. A‐B, Screening and validation of the soft threshold. Four was chosen as the soft threshold for further analysis. C, Clustering dendrogram of genes in EC tissues. D, Correlation between modules and risk model and identification of the key module. E, Enrichment analysis in the Metascape database and the top 20 enrichment terms were shown. F, Network of the enrichment terms

For a better understanding the relationship between the risk score model and molecular groups, we extracted RNA‐seq data and conducted WGCNA. We selected the top 25% DEGs and obtained 5155 mRNA between normal and cancer samples. One of the most critical parameter was power value, which mainly affected the independence and the average connectivity degree of co‐expression modules. The soft threshold for network construction was selected as 4 (Figure 4A). Meanwhile, the fitting degree of scale‐free topological model was 0.9. Thus, this network conformed to the power‐law distribution and was closer to the real biological network state (Figure 4B). These co‐expression modules were then constructed and divided into 10 meaningful modules (Figure 4C). By analyzing the associations between the gene modules and risk score, we found that the green module had the highest correlation (Cor = 0.56, *P* = 7e‐12 for risk; Cor = 0.49, *P* = 9e‐10 for risk score) with the five‐DNA methylation signature (Figure 4D). There were 263 genes in the green module and we then analyzed these genes with Metascape. The Metascape analysis shows the top 20 clusters of functional enriched sets (Figure 4E). Based on the literature, cell surface receptor signaling pathway involved in cell–cell signaling (GO: 1905114), epithelial to mesenchymal transition (EMT, GO: 0042476), cellular response to growth factor stimulus (GO: 0071363), positive regulation of MAPK cascade (GO: 0043410) were the four terms associated with the biological mechanisms of EC.^18^, ^19^ These were the four terms we were interested in. According to the data processing method described on Metascape, terms with a similarity score >0.3 were linked by an edge, forming a clustered network (Figure 4F). In this network, each term was represented by a circle node, where its size was proportional according to the number of input genes that fell into that term, and the color represented its cluster identity. We selected the clusters containing the previous four terms and named them as the key clusters due to the similarity of terms in the same cluster. These results revealed that the five‐DNA methylation risk model was significantly associated with a functional gene module, which might work through EMT‐ and MAPK‐signaling pathway.

### 3.5 Development and evaluation of the nomogram for OS in EC

**FIGURE 5:**
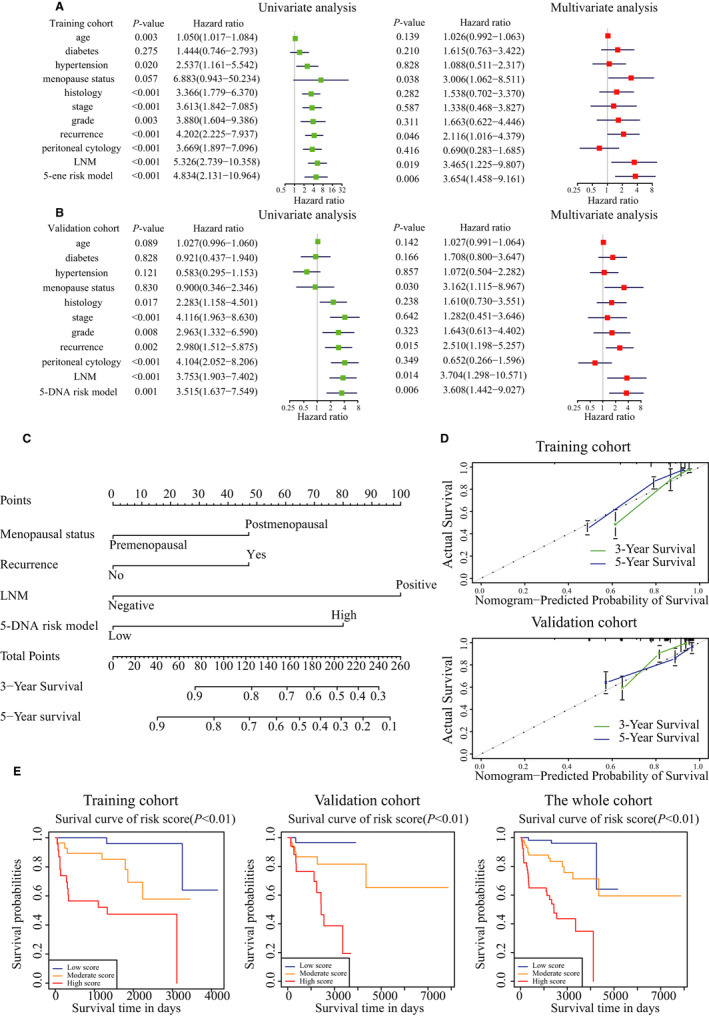
Establishment of a nomogram for survival prediction. Univariate and multivariate analyses of the association between clinicopathological factors (including the risk score model) and overall survival of EC patients in A, training cohort and B, validation cohort. The hazard ratios (HR), 95% confidence intervals. C, Nomogram including the 5‐DNA methylation risk signature and clinicopathological features. D, Calibration plot of the nomogram‐predicted probability and actual survival in training and validation cohorts. E, Kaplan–Meier survival analysis of the three subgroups. F, Time dependent ROC curves for a risk score model and several complete clinicopathological information of EC patients

To provide the clinician with a quantitative method by which to predict a patient's probability of OS, a nomogram that integrated the risk score model and clinicopathological characteristics was developed. The whole patients in TCGA database was evenly divided into two cohorts, the training cohort and the validation cohort, randomly. We then conducted univariate and multivariate regression analyses in the training cohorts, and the results suggested that menopausal status (OR = 3.006, 95%CI: 1.062–8.511, *p* = 0.038), recurrence (OR = 2.116, 95%CI: 1.016–4.379, *p* = 0.046), LNM (OR = 3.465, 95%CI: 1.225–9.807, *p* = 0.019), and five‐DNA methylation risk model (OR = 3.654, 95%CI: 1.458–9.161, *p* = 0.006) were identified as the four independent risk factors (Figure 5A). The validation group showed the same results (Figure 5B). Next, we constructed the nomogram utilizing the independent risk factors (menopausal status, recurrence, LNM, five‐DNA methylation risk model) from the multivariate analysis in the training cohort (Figure 5C). Concordance index (C‐index) of the nomogram was 0.815 (95%CI: 0.774–0.920) in the training cohort and 0.802 (95%CI: 0.762–0.903) in the validation cohort. The prediction efficiency of 3‐year survival and 5‐year survival in training and validation groups were confirmed by the calibration (Figure 5D). According to the nomogram, the variables were assigned with a corresponding score (Table 3) so that we calculated the total score of each patient. We then categorized the patients into three subgroups as low‐, moderate‐, and high score subgroups in the basis of total score. The Kaplan–Meier curve indicated that the OS of the three subgroups was significantly different from each other in the training, validation, and the whole cohorts (all *p* < 0.01, Figure 5E). To further explore the efficiency of the risk model in the nomogram, we conducted the ROC curve analysis and compared the AUCs between the three‐factor group (menopausal status, recurrence, LNM) and the three‐factor + five‐DNA methylation risk signature in the training cohort, validation cohort, and the whole cohort (Figure S4A–C). We further validated the nomogram in our 24 patients. We calculated the total score of each patient and divided the 24 patients into two groups according to the nomogram. Kaplan–Meier survival curve indicated that patients in two groups had significantly different OS (*p* < 0.05, Figure S5). The results revealed that after adding the risk signature, AUC of the nomogram was significantly increased in all of the three cohorts (for training cohort: 0.745 vs 0.828, for validation cohort: 0.781 vs 0.866, for the whole cohort: 0.749 vs 0.843, all *p* value <0.05).

**TABLE 3:** Corresponding risk score for each variable and total score

Variables	Score
Menopausal status	
Premenopausal	0
Postmenopausal	47.5
Recurrence	
No	0
Yes	47.5
LNM	
Negative	0
Positive	100
Risk signature	
Low	0
High	80
Total score	
Low risk	0–47.5
Moderate risk	95–147.5
High risk	>147.5

Abbreviation: LNM, lymph node metastasis.

### 3.6 Prognostic value of the nomogram in different clinicopathological subgroups

**FIGURE 6:**
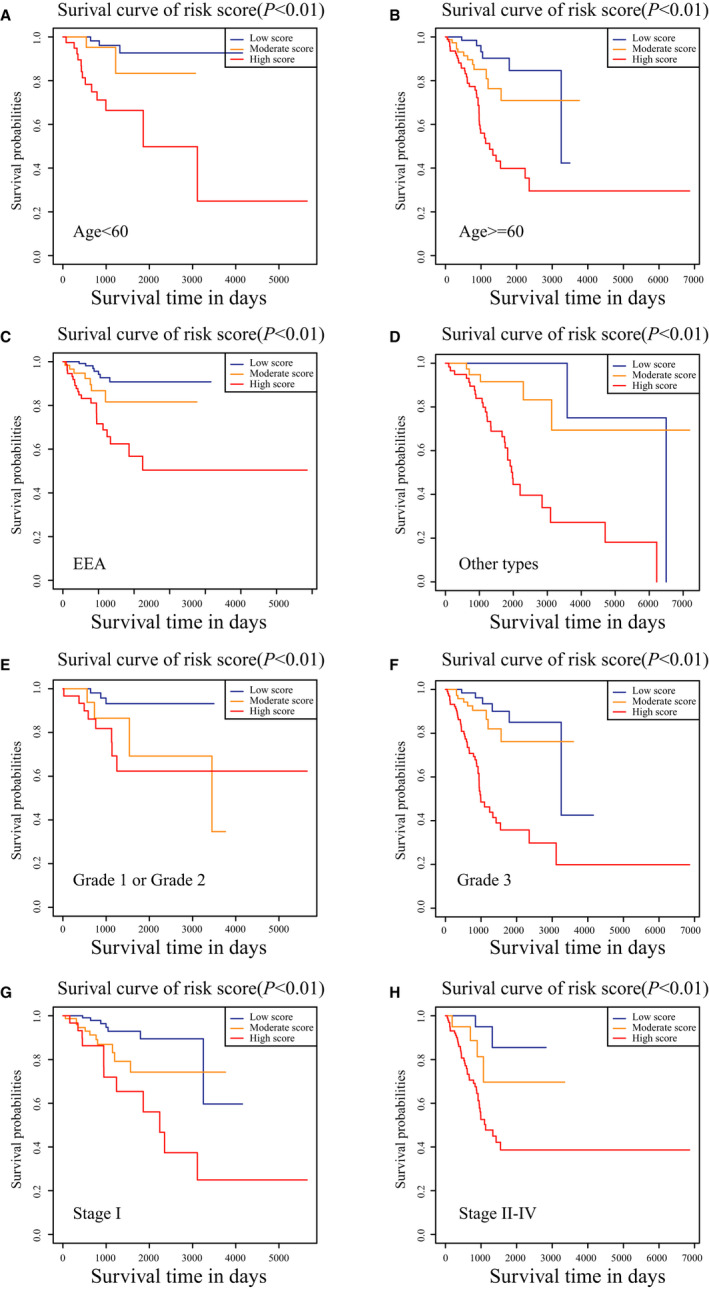
Prognostic value of the nomogram in different clinicopathological subgroups. A, Age < 60 years old. B, Age ≥ 60 years old. C, EEA. D, Other types. E, Grade 1 and grade 2. F, Grade 3. G, FIGO stage I. H, FIGO stage II–IV

To further evaluate and test the survival assessment model, we conducted Kaplan–Meier survival analysis in different clinicopathological subgroups (age subgroups: age < 60 and age ≥ 60; histological type subgroups: EEA and other types; tumor grade subgroups: Grade 1/2 and Grade 3; clinical stage subgroups: stage I and stage II–IV) in the whole patients. The tests showed that the predictive capability of the survival assessment model was effective in all of the clinicopathological subgroups for EC patients (Figure 6A–H). Thus, the model had a certain reliability and practicability in evaluating prognosis.

## 4 DISCUSSION

During the past 20 years, the mortality of endometrial cancer (EC) mortality had increased by more than 100%.^20^ EC had been reported to be the most common female genital malignant tumor in western country.^2^ Histological type and FIGO staging system is a key factor in current clinical decision making and prognosis of EC. However, EC patients with identical histological type or FIGO stage received the same therapy might have significantly different clinical outcomes, indicating that the current classification system was not sufficient to predict the prognosis of patients. Therefore, it was imperative to reveal the pathogenic mechanism of EC and provide effective intervention to guide individualized treatment and improve the therapeutic effect. About 100 post‐transcriptional chemical modifications occur in biological RNAs^21^ and methylation had become one of the most abundant endochemical modifications. In recent decades, researchers had found that aberrant DNA methylation was characteristics of many cancers and often occurred as an early event in tumorigenesis.^22^, ^23^

There are two kinds of values representing methylated level, beta‐value, and M‐value. The relationship between the beta‐value and M‐value is a logit transformation. Beta‐value is the most widely used in quantifying methylation level and mainly used for differentially analysis, and M‐value commonly used for the comparison of characteristics between samples. Du et al.^24^ mentioned that the M‐value is more statistically valid for the differential analysis of methylation levels, many recent researches still use beta value for differential analysis. What's more, the manufacturer recommended beta‐value method for analyzing Illumina Infinium HumanMethylation BeadChip microarrays. Therefore, we choose beta‐value for further analysis. In this study, we comprehensively analyzed the profiling of DNA methylation in a cohort of EC patients with different OS from the TCGA database to investigate altered DNA methylation patterns. Additionally, five methylated DNA (GBP4, OR8K3, GABRA2, RIPPLY2, and TRBV5‐7) were screened by LASSO regression and their expressions were also validated in the database. Among all these 5 DNA, GBP4, GABRA2, and RIPPLY2 were once reported in cancer research. GBP4 methylation was high in cancer tissues in EC, which indicated that GBP4 was a favorable biomarker in EC. Data in our center proved this result. Studies in other cancers also confirmed our results. The expression of GBP4 could improve the sensitivity and specificity for predicting skin cutaneous melanoma.^25^ In another study, correlation between GBP4 and 5‐year survival rate was favorable in colorectal cancer and breast cancer.^26^ GABRA2 was a cervical cancer‐specific marker and could be used for diagnosis, which may boost the development of new epigenetic therapies.^27^ RIPPLY played a pivotal role in mammalian embryogenesis and tumorigenesis, and the downstream molecule of RIPPLY often targeted at WNT3A pathway.^28^ Furthermore, risk score according to the coefficient and expression of each patient was calculated and divided into two groups based on the median risk score of cut‐off point. Afterwards, a prognostic risk model based on the five DNA was established. The model could distinguish difference in OS between the two risk groups based on the Kaplan–Meier curves, which could help to determine credible individual measures for the patients. The five‐DNA methylation risk model also had a good performance in the prediction of OS by tdROC analysis. The risk model was further brought into a multivariate analysis, and a nomogram consisting of risk signature and clinicopatholgical factors was constructed.

Recent studies revealed an eight‐DNA methylation signature that could predict the prognosis of EC by the GEO‐based bioinformatics analysis.^29^ In another study, researchers had identified a new methylated risk signature, which could be a useful marker for distinguishing tumors and normal tissues.^30^ Ying et al. found a 15‐CpG marker and it had shown to have a higher discriminative ability to distinguish EC patients with an elevated risk of mortality than the FIGO staging system and several other clinical prognostic variables.^31^ Mutations and methylation of mismatch repair genes also played a pivotal role in EC.^32^ Currently, several biomarkers for EC prognosis were available. Hsu et al. found that hypomethylated signatures of candidate BMP genes associated with EpCAM‐mediated expression present putative biomarkers predictive of poor survival in EC.^33^ However, most of the established model for therapy concentrated on the single biomarker, leading to the inadequate efficiency of their risk signature. Multiple‐biomarker models for therapy response and survival of EC were seldom based on clinical traits, thus limiting corresponding accuracy and specificity. Our model not only identified a multi‐DNA model but combined the model with clinicopathological characteristics as well. GSEA results found that risk factors triggering the five dysregulated genes are enriched in tight junction (TJ) and ECM receptor interaction. TJ was regulated by estrogen and the expression of TJ decreased with the elevation of estrogen, resulting in promotion of the proliferative, migratory, and invasive capabilities of EC cells.^34^ Extracellular matrix (ECM) was a non‐cellular three‐dimensional macromolecular network composed of collagens, proteoglycans, elastin, fibronectin, laminins, and several other glycoproteins.^35^ Receptors in ECM such as integrin and TGF‐β regulated diverse cellular functions, including survival, growth, migration, and invasion, and are essential for maintaining normal homeostasis.^36^ These ECM receptors could also induce the epithelial‐mesenchymal transition (EMT), which often resulted from the induction of transcription factors that altered gene expression to promote loss of cell–cell adhesion, leading to a shift in cytoskeletal dynamics and change from epithelial morphology to the mesenchymal phenotype.^37^ The promotion of MAPK pathway enhanced metastasis and EMT in cancer,^38^, ^39^ which was revealed by the integrated study of Metascape and WGCNA in our study. Above all, these results indicated that high‐risk group in the model had a poor prognosis and were more likely to become invasive or metastatic EC.

As the efficacy of any single biomarker was inadequate, our study developed a multiple‐risk signature combined the risk score model and clinicopathological characteristics together to increase the prognostic value of OS in EC patients. Multivariate analysis in the training cohort identified menopausal status, recurrence, LNM, and risk model as the independent risk factors and these factors were used to establish a nomogram. The predicted survival rate of the nomogram was close to the actual survival situation (C‐index: 0.815 and 0.802 for the training cohort and validation cohorts, respectively). AUC was significantly different between groups with or without the risk score model in the training cohort, validation cohort, and the whole population (all *p* < 0.01). Furthermore, we internally and externally validated the nomogram in different pathological stratifications to illustrate its stability and reliability. The results indicated an excellent predictive ability of this nomogram in the prognosis of EC patients.

Previous studies also constructed nomograms for predicting survival of EC patients. The most famous and widely used nomogram was developed by Memorial Sloan Kettering Cancer Center (MSKCC).^40^ MSKCC nomogram consisted of five clinicopathological factors including age, lymph node status, FIGO stage, tumor grade, and histological subtype, which showed better discrimination and calibration values than the FIGO staging system^41^ and could better stratified patients in other external validation.^42^ There were also molecule‐based nomograms predicting OS of EC.^43^, ^44^ However, these studies only concentrated on either clinical indexes or gene expression, and the AUCs of these nomograms of 5‐year survival were lower than ours (AUC = 0.866). Overall, our risk signature and nomogram could enable doctors to identify high‐ and low‐risk EC patients, delivering helpful evidences to make better individualized treatment.

To the best of our knowledge, the five‐DNA methylation risk model had never been previously reported, and the nomogram that combined methylated DNA information and clinicopathological factors would help clinicians to identify new prognostic biomarkers in EC in both a clinical and a basic perspective.

However, there are several limitations in our study. First, this is a single‐center study, although the application of this risk model is verified by external validation, our data only conduct the RNAseq but not methylated expression. A large sample size is needed for further validation. Second, our study focus on the methylated DNA in the database, and there are many other modified formations in cancer. It will be of significance to integrate all these modifications together to study tumorigenesis and progression in EC.

## 5 CONCLUSION

Above all, our study revealed a five‐DNA risk model based on methylation profiling. The model indicated that methylation detection might be an important means to help establish a new evaluation system for prognosis and acted as a therapeutic target for individualized treatment. Further nomogram facilitated the clinicians an easy way to diagnose and predict the prognosis of EC patients.

## CONFLICT OF INTERESTS

The authors have no conflicts of interest to declare.

## AUTHORS' CONTRIBUTIONS

XL and YC conceived and designed the experiments. ZW and JZ downloaded and collected the data. FY and YF analyzed the RNA sequencing data. XL and YD analyzed the data. XL wrote the paper. JW and XL reviewed the draft. All authors read and approved the final manuscript.

## ETHICS APPROVAL AND CONSENT TO PARTICIPATE

This study was approved by the Institutional Ethics Committee (Human Research) of Peking University People's Hospital.

## CONSENT FOR PUBLICATION

All the authors have read and approved the final manuscript.

## Supporting information

Fig S1

Fig S2

Fig S3

Fig S4

Fig S5

## Data Availability

The database supporting the conclusions of this article is available in the TCGA database and GEO database.
