# Supplementary figures and images for: Establishment and validation of a prognostic nomogram based on a novel five‐DNA methylation signature for survival in endometrial cancer patients
Source: Cancer Med. 2020 Dec 22;10(2):693–708. doi: 10.1002/cam4.3576 (PMC7877372; doi:10.1002/cam4.3576)

**A**Cor=-0.483( $P$ -value=1.909e-10)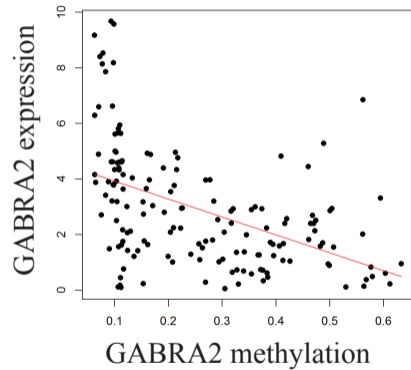**B**Cor=-0.301( $P$ -value=1.788e-4)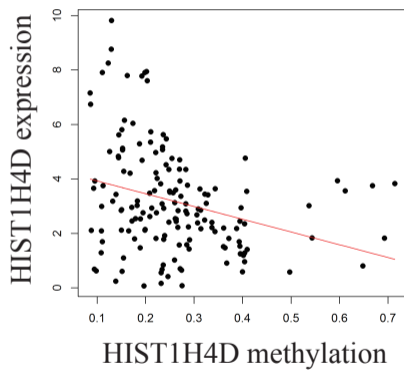**C**Cor=-0.439( $P$ -value=1.125e-8)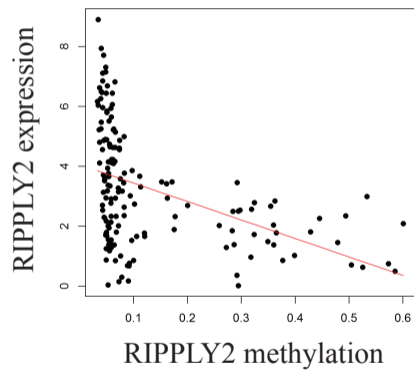

Supplement: Supplementary file 1 — Fig S1 [file CAM4-10-693-s001.pdf]

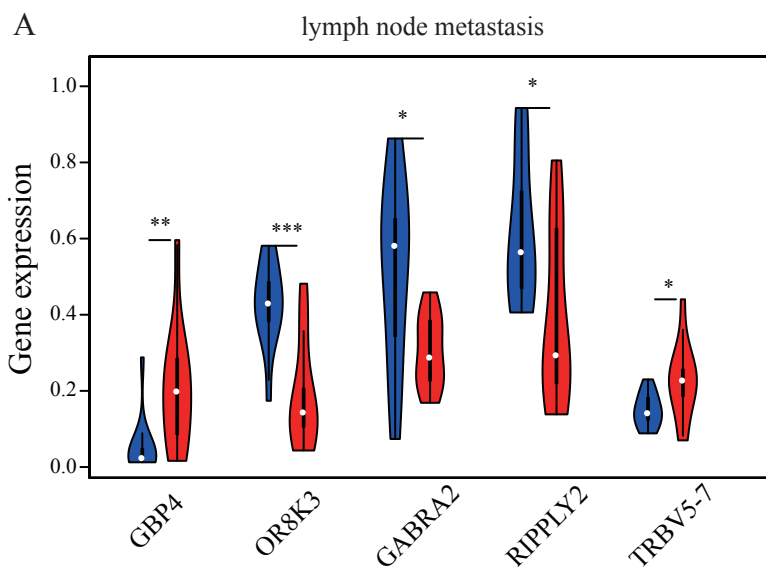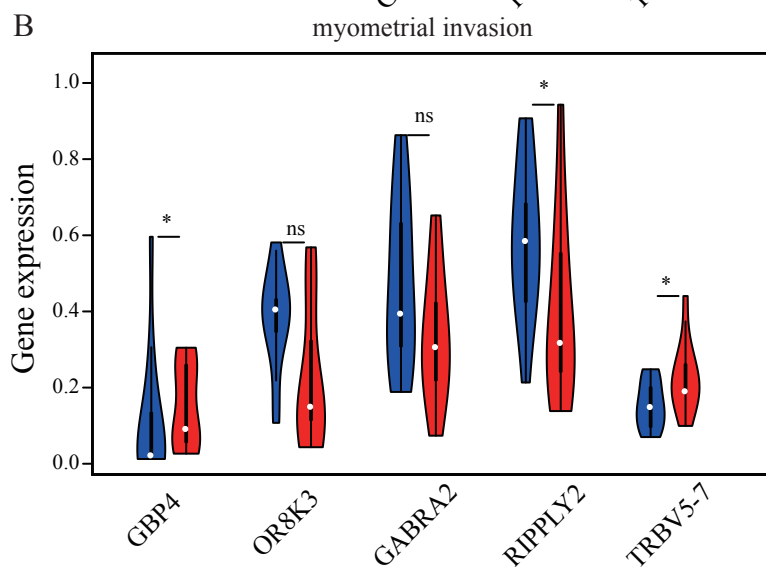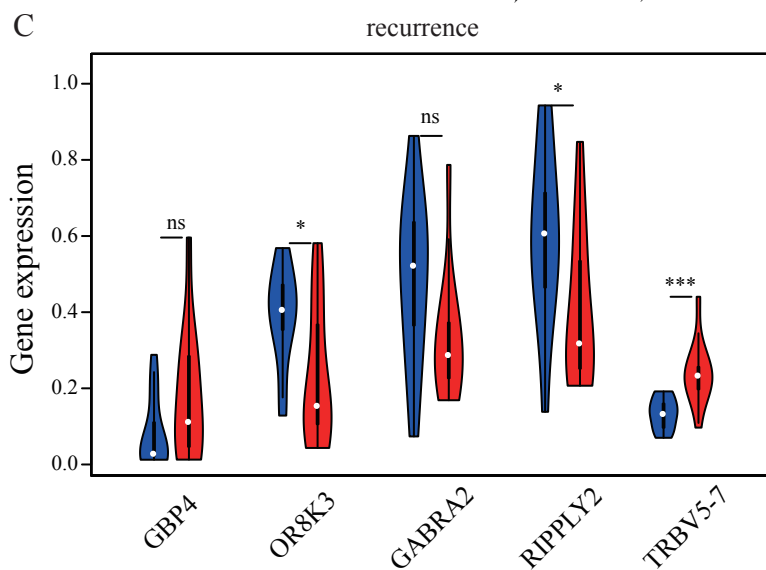

Supplement: Supplementary file 2 — Fig S2 [file CAM4-10-693-s002.pdf]

**A**

ROC curve in training cohort

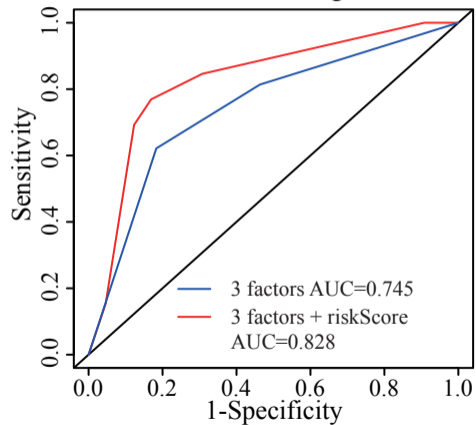**B**

ROC curve in validation cohort

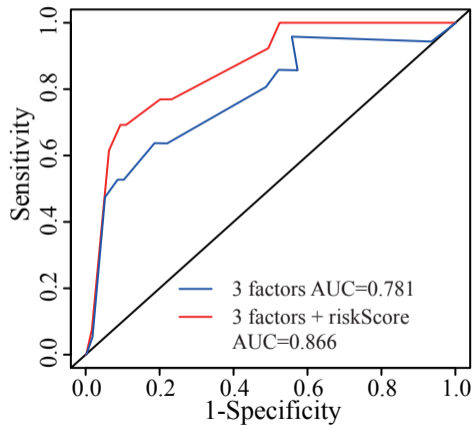**C**

ROC curve in the whole cohort

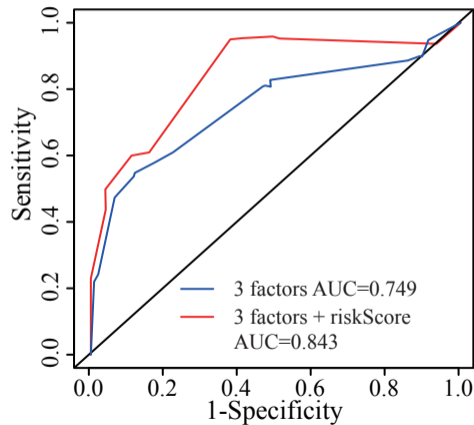

Supplement: Supplementary file 4 — Fig S4 [file CAM4-10-693-s004.pdf]

Survival curve of risk score( $P=0.0235$ )

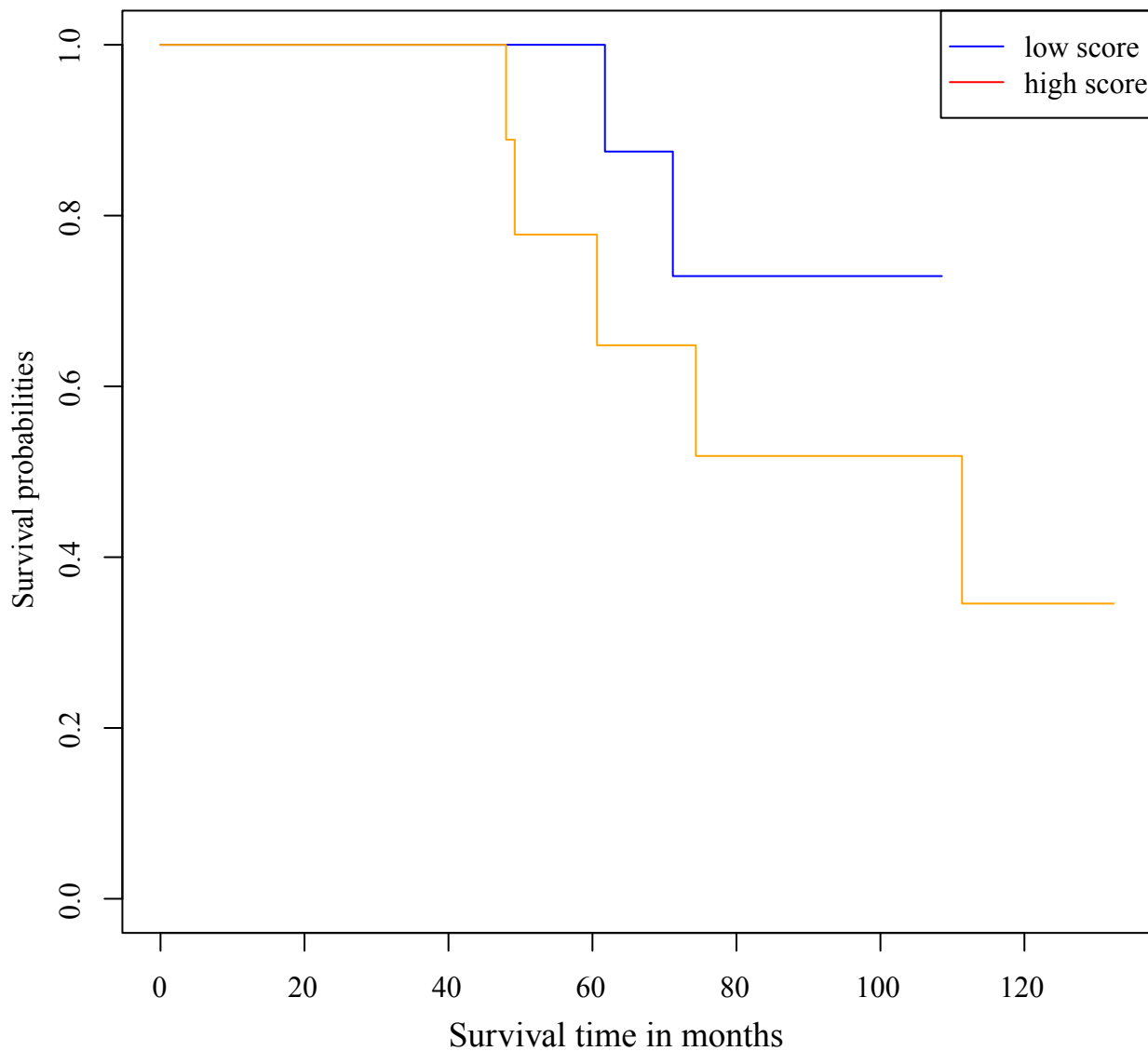

Supplement: Supplementary file 5 — Fig S5 [file CAM4-10-693-s005.pdf]
