# Supplementary material for: Establishment and validation of a prognostic nomogram based on a novel five‐DNA methylation signature for survival in endometrial cancer patients
Source: Cancer Med. 2020 Dec 22;10(2):693–708. doi: 10.1002/cam4.3576 (PMC7877372; doi:10.1002/cam4.3576)
Supplement: Supplementary file 3 — Fig S3 [file CAM4-10-693-s003.pdf]

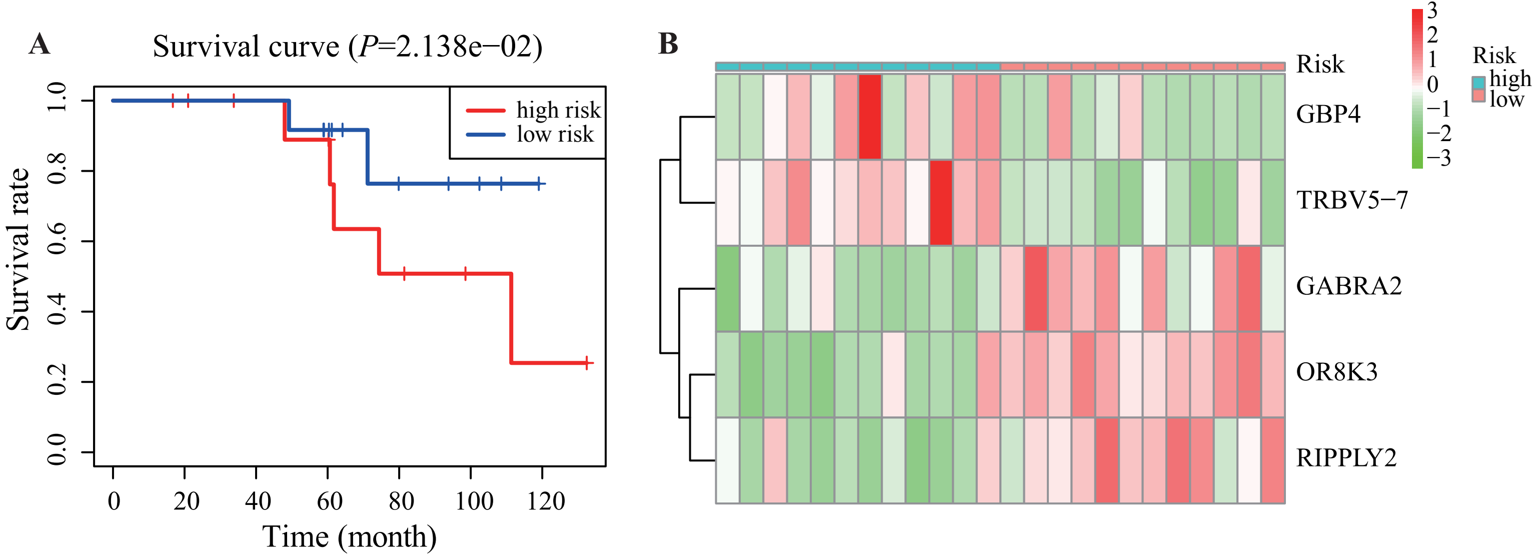
A
Survival curve (P=2.138e−02)
high risk
low risk
Survival rate
0.0
0.2
0.4
0.6
0.8
1.0
0
20
40
60
80
100
120
Time (month)
B
Risk
GBP4
TRBV5−7
GABRA2
OR8K3
RIPPLY2
3
2
1
0
−1
−2
−3
Risk
high
low
